# Mitochondrial Molecular Abnormalities Revealed by Proteomic Analysis of Hippocampal Organelles of Mice Triple Transgenic for Alzheimer Disease

**DOI:** 10.3389/fnmol.2018.00074

**Published:** 2018-03-09

**Authors:** Haitao Yu, Xuemei Lin, Dian Wang, Zaijun Zhang, Yi Guo, Xiaohu Ren, Benhong Xu, Jianhui Yuan, Jianjun Liu, Peter S. Spencer, Jian-Zhi Wang, Xifei Yang

**Affiliations:** ^1^Key Laboratory of Modern Toxicology of Shenzhen, Institute of Toxicology, Shenzhen Center for Disease Control and Prevention, Shenzhen, China; ^2^Institute of New Drug Research and Guangzhou, Key Laboratory of Innovative Chemical Drug Research in Cardio-Cerebrovascular Diseases, Jinan University College of Pharmacy, Guangzhou, China; ^3^Department of Neurology, Second Clinical College, Jinan University, Shenzhen, China; ^4^Department of Neurology, School of Medicine and Oregon Institute of Occupational Health Sciences, Oregon Health and Science University, Portland, OR, United States; ^5^Department of Pathophysiology, School of Basic Medicine and the Collaborative Innovation Center for Brain Science, Key Laboratory of Ministry of Education of China and Hubei Province for Neurological Disorders, Tongji Medical College, Huazhong University of Science and Technology, Wuhan, China

**Keywords:** Alzheimer’s disease (AD), mitochondrial/nuclear proteomics, biomarkers

## Abstract

Mitochondrial dysfunction is implicated in the pathogenesis of Alzheimer’s disease (AD). However, the precise mitochondrial molecular deficits in AD remain poorly understood. Mitochondrial and nuclear proteomic analysis in mature male triple transgenic AD mice (PS1M146V/APPSwe/TauP301L) by two-dimensional fluorescence difference gel electrophoresis (2D-DIGE) coupled with MALDI-TOF-MS/MS, bio-informatics analysis and immunofluorescent staining were performed in this study. In addition to impaired spatial memory impairment and intracellular accumulation of amyloid 1–42 (Aβ_1–42_) in the 3xTg-AD mice, a well-accepted mouse model of the human disease, we also found significantly increased DNA oxidative damage in entorhinal cortex, hippocampal CA1, CA3 and dental gyrus (DG), as evidenced by the positive staining of 8-hydroxyguanosine, a biomarker of mild cognitive impairment early in AD. We identified significant differences in 27 hippocampal mitochondrial proteins (11 increased and 16 decreased), and 37 hippocampal nuclear proteins (12 increased and 25 decreased) in 3xTg-AD mice compared with the wild-type (WT) mice. Differentially expressed mitochondrial and nuclear proteins were mainly involved in energy metabolism (>55%), synapses, DNA damage, apoptosis and oxidative stress. Two proteins were differentially expressed in both hippocampal mitochondria and nuclei, namely electron transport chain (ETC)-related protein ATP synthase subunit d (ATP5H) was significantly decreased, and apoptosis-related dynamin-1 (DYN1), a pre-synaptic and mitochondrial division-regulated protein that was significantly increased. In sum, perturbations of hippocampus mitochondrial energy metabolism-related proteins responsible for ATP generation via oxidation phosphorylation (OXPHOS), especially nuclear-encoded OXPHOS proteins, correlated with the amyloid-associated cognitive deficits of this murine AD model. The molecular changes in respiratory chain-related proteins and DYN1 may represent novel biomarkers of AD.

## Introduction

Alzheimer’s disease (AD) is a common neurodegenerative disorder characterized by an age-inappropriate decline in memory, language, thinking and judgment. The main pathological features of AD include beta-amyloid (Aβ) deposition leading to senile plaques, tau protein hyperphosphorylation, neurofibrillary tangles and early synaptic loss, evolving to axonal and neuronal degeneration (Maccioni et al., 2001). Accumulation of tau protein can cause an abnormal distribution of mitochondria within neurons (Kopeikina et al., 2011) and buildup of Aβ and APP on mitochondrial membranes can result in structural and functional damage to these critically important organelles (Pagani and Eckert, 2011; Rosales-Corral et al., 2012; Pinho et al., 2014).

Mitochondrial dysfunction of neural tissue is closely related to the occurrence and development of AD, as well as other neurodegenerative diseases such as Parkinson and Huntington diseases (Kopeikina et al., 2011). Cellular apoptosis, mitochondrial dysfunction and dysfunctional energy metabolism are early pathological features of these diseases (Tatsuta and Langer, 2008; Pathania et al., 2009). Mitochondrial dysfunction mainly manifests as disordered energy metabolism, perturbations in the electron transport chain (ETC), synaptic dysfunction and neuronal apoptosis (Du and Yan, 2010; Müller et al., 2010). Dysfunction of the mitochondrial ETC results in reduced ATP synthesis, generation of free radicals, and oxidative damage resulting in neuronal dysfunction (Guzowski and McGaugh, 1997; Chan, 2006a). Changes in the dynamics of mitochondria, notably increased organelle fission, may also contribute to a number of neurodegenerative disorders (Blass et al., 2000; Atamna and Frey, 2007; Reddy and Beal, 2008). A dynamin-associated protein Drp1 that induces mitochondrial fission and increases the lifespan of *Drosophila melanogaster* (Rana et al., 2017) has potential relevance to healthy aging and longevity of humans (Rana et al., 2017). While mitochondrial dysfunction clearly plays an important role in the pathogenesis of AD, we know little about the underlying molecular mechanisms.

To illuminate molecular mechanisms and find new biomarkers of AD, we have used mature 3xTg-AD mice to investigate the hippocampal mitochondrial and nuclear proteome by two-dimensional fluorescence difference gel electrophoresis (2D-DIGE) coupled with MALDI-TOF-MS/MS.

## Materials and Methods

### Animals and Treatment Protocol

Triple transgenic AD male mice (3xTg-AD) harboring the human mutation of APP_Swe_, PS1_M146V_ and Tau_P301L_ (strain: B6; 129-Psen1^tm1Mpm^ Tg [APPSwe, tauP301L] 1Lfa/Mmjax), plus wild-type (WT) mice (strain: B6129SF2/J) from the same genetic background were purchased from Jackson Laboratory (Maine, USA). All animal experiments were conducted in accord with the Principles of Laboratory Animal Care (NIH publication No. 8-23, revised 1985) and the Regulations of the Animal Care and Use Committee of the Experimental Animal Center of the Shenzhen Center for Disease Control and Prevention. This animal study was approved by The Ethics Committee of Shenzhen Center for Disease Control and Prevention. The least number of animals was used in experimentation, and efforts were made to minimize animal discomfort. Mice were housed in groups of 10 animals per cage (470 × 350 × 200 mm) and maintained on a 12-h light-dark cycle with food and water available *ad libitum*. The animal-housing room was maintained at stable temperature (24 ± 2°C) and relative humidity (55 ± 5%).

### Morris Water Maze Test

A Morris Water Maze was used to assess spatial learning and memory of mice (Guzowski and McGaugh, 1997; D’Hooge and De Deyn, 2001; Matsushima et al., 2012). During the experiment, the investigator did not know the experimental grouping. Mice were transferred to the test room at least 1 h in advance to acclimate to the environment. Mice were trained for five consecutive days to find a submerged platform (2 cm below the water surface) hidden in the water maze. Water temperature was maintained at 25 ± 2°C. During each trial, mice were placed in the middle of one of the four quadrants while facing the wall of the pool. Training ended when the animal reached the platform. If an animal did not find the platform within 60 s of placement in the quadrant, they were guided to the platform and kept there for 30 s. Long-term memory was evaluated 6 days after training completion. After removing the platform, mice were allowed to swim in the water for 120 s to find the previous platform location. All water maze test parameters, such as the percentage of time spent and the percentage of distance traveled in the target quadrants, were recorded with a video-image analysis system.

### Isolation of Mitochondria

After the behavioral tests had been completed, animals were euthanized with diethyl ether, the brain removed from the skull, and the hippocampus excised. Mitochondria were isolated at 4°C using the Mitochondria Isolation Kit for Tissue (Thermo 89801, Thermo Fisher Scientific, Waltham, MA, USA). Hippocampal tissue containing the dentate gyrus (DG) and CA1 zone was homogenized for 10 min in 300 μL 1× PBS solution with a Motor Driven Tissue Grinder (Sangon Biotech G506003, Shanghai, China). Homogenates were centrifuged at 1000 *g* for 5 min at 4°C and the resulting pellet suspended in 800 μL of BSA/Reagent A solution and incubated on ice for exactly 2 min. Ten microliter of Mitochondria Isolation Reagent B were added to the suspension and the sample incubated on ice for 5 min. Then, 800 μL of Mitochondria Isolation Reagent C was added to the tube and mixed. Finally, the mitochondrial pellet was isolated after centrifuging, washed twice with 500 μL of Wash Buffer, and stored at −80°C until used.

### Isolation of Nuclei

Nuclear material was isolated with NE-PER Nuclear and Cytoplasmic Extraction Reagents (ThermoFisher 78835). Bilateral hippocampal tissues were homogenized in 300 μL Cytoplasmic Extraction Reagent I (CER I) for 10 min with the aid of a Motor-Driven Tissue Grinder (Sangon Biotech G506003, Shanghai, China). Seventeen microliters of ice-cold Cytoplasmic Extraction Reagent II (CERII) were added to the swollen cells to gently lyse the cell membrane. Then, 150 μL ice-cold Nuclear Extraction Reagent (NER) were used to extract nuclear proteins from the pellet. The sample was placed on ice and vortexed for 15 s every 10 min, with four repeats. The nuclear extract was transferred to a clean pre-chilled tube and stored at −80°C until use.

### Protein Sample Preparation

To extract proteins for differential in-gel electrophoresis (DIGE) analysis, DIGE-specific lysis buffer (7 M urea, 2 M thiourea, 30 mM Tris-HCl, 4% CHAPS, pH 8.5) was used to treat the mitochondrial pellets and nuclear extracts of whole hippocampus. Pellets were incubated for 30 min, centrifuged at 20,000 *g* for 60 min at 4°C, and then at 15,000 *g* for 30 min at 4°C, to remove salt and other impurities. Protein concentrations were determined by 2-D Quant Kit (GE HealthCare, Milwaukee, WI, USA), and protein solutions were stored at −80°C until use.

### DIGE Labeling of Proteins

CyDye (GE Healthcare) powder was dissolved at a concentration of 1 nmol/μL with 5 μL of 99.8% anhydrous N,N-dimethylformamide (DMF, 227056, Sigma-Aldrich, St. Louis, MO, USA), followed by dilution of the stock solution with DMF to a final concentration of 200 pmol/μL. A total of 25 μg of each protein sample held at pH 8.0–9.0 was labeled with 200 pmol of either Cy3 dyes (GE Healthcare, 25-8008-61), or Cy5 dyes (GE Healthcare, 25-8008-62). In addition, a mixed sample (25 μg each) labeled with Cy2 dyes was used as an internal standard. The labeled reactants were incubated on ice for 30 min in the dark, and the reaction was terminated with 10 mM lysine (Sigma-Aldrich, L5626) at 4°C for 10 min in the dark. After labeling, the Cy2-, Cy3- and Cy5-labeled samples were mixed together. IPG buffer (2% (v/v) pH 3–11 NL), 100 μL of 2× lysis buffer (7 M urea, 2 M thiourea, 4% CHAPS, 2% DTT) was added and the samples incubated on ice for 10 min. Finally, rehydration buffer was added to adjust the volume to 450 μL.

### 2D Difference Gel Electrophoresis (2D-DIGE)

The first dimension was performed with an Ettan IPGphor Isoelectric Focusing System (GE Healthcare). Mixtures of Cy2-, Cy3- and Cy5-labeled proteins were put into 24 cm pH 3–11 NL Immobiline DryStrips (GE Healthcare). Specific steps, carried out at RT, were as follows: 300 V 12 h; 500 V 2 h; 1000 V 2 h; gradient 8000 V 8 h; 8000 V 8 h. After IEF, each strip was equilibrated in equilibration buffer (6 M urea, 75 mM Tris-HCl, 30% glycerol, 2% SDS), followed by 1% DTT (Sigma-Aldrich) and 4.5% IAA (Sigma-Aldrich) for 15 min. The equilibrated strips were loaded on the top of 12.5% SDS-PAGE gels with 0.5% (w/v) ultra-low-melting-point agarose sealing solution (25 mM Tris, 192 mM glycine, 0.1% SDS, 0.5% (w/v) agarose, 0.02% bromophenol blue). Electrophoresis was performed with an Ettan DALTsix Electrophoresis System (GE Healthcare) under the following conditions: 1 W/gel for 1 h and, subsequently 11 W/gel for 5 h at 12°C in darkness. Gels were immediately scanned with a Typhoon TRIO Variable Mode Imager (GE Healthcare), and the PMT was set to ensure that the maximum pixel intensity of all gel images remained within a range of 40,000–60,000 pixels.

### Image Analysis

The DeCyder package (version 6.5, GE Healthcare) was used to analyze DIGE gels for protein relative quantification according to the manufacturer’s protocol. For each protein spot, the spot volume was determined in the Cy5 or Cy3 channels and then normalized according to the corresponding Cy2 spot volume. The normalized spot volume was compared across gels and among the replicate groups. Spots with a significant difference (*p* < 0.05) between 3xTg-AD and WT were further analyzed.

### Spot Picking and In-Gel Digestion

We used 1.5 mg of protein samples to run 2D-DIGE under the conditions described above. Gels were stained with Coomassie blue solution containing 0.12% Coomassie Brilliant Blue G-250, 20% ethanol, 10% phosphoric acid, and 10% ammonia sulfate. Spots of interest detected by Decyder software analysis were manually excised from the stained gel and digested overnight at 37°C with trypsin (Promega Corp., WI, USA). The tryptic peptides were used for MALDI-TOF-MS/MS analysis.

### Mass Spectrometry

Peptide analysis was performed with a MALDI-TOF/TOF 5800 mass spectrometer system (AB SCIEX, Framingham, MA, USA). Peptide extracts (0.6 μl) were crystallized (with 0.4 mg/mL α-cyano-4-hydroxycinnamic acid in 30% acetonitrile/0.06% trifluoroacetic acid) and dried directly on the target. The spectra were externally calibrated. Mitochondrial and nuclear proteins were identified with the SwissProt *Mus musculus* database housed in MASCOT (Matrix Science, London, UK). The search was conducted with a tolerance on mass measurement of 100 ppm in the MS mode and 0.5 Da in the MS/MS mode. The fixed carbamidomethyl modification was taken into account, and up to two missed cleavages per peptide were allowed.

### Bioinformatics Analysis

DAVID version 6.7^1^ was used to elucidate the biological functions of identified proteins (Huang da et al., 2009a,b). For protein-protein interaction network analysis, the differently regulated proteins were subjected to STRING database version 10.0^2^ (Xu et al., 2016). The STRING-generated network is visualized and edited in Cytoscape version 3.5.1. A Venn Diagram Generator^3^ was used to perform a logistic analysis of the mitochondrial proteome and nuclear proteome. Cytoscape 3.5.1 software and plug-ins were used to analyze WikiPathways (Xu et al., 2016).

### Immunofluorescence Staining

Brain tissues were fixed for 48 h in 4% paraformaldehyde at 4°C, dehydrated in ethanol and embedded in paraffin. Coronal sections of the hippocampus were obtained to analyze Aβ_1–42_ protein and DNA oxidative damage in the form of 8-hydroxydeoxyguanosine (8-OHdG) DNA adducts, After dewaxing and rehydration, the sections were treated with 0.01 M citrate buffer (pH 6.0) and 0.1% Tween-20 at 90–95°C for 5 min for antigen retrieval. Tissues were then incubated at 4°C overnight with the primary antibody to 8-OHdG (1:200, Abcam, ab10802) and to Aβ_1–42_ (1:100, Abcam, ab10148). After washing with PBS, tissues were stained for 1 h in darkness at RT with the secondary antibody, namely FITC-goat anti-rabbit IgG (H + L), and then counterstained with DAPI (Beyotime Institute of Biotechnology, Haimen, Jiangsu, China) for 1 min to reveal the nuclei, Tissues were examined and images taken with a laser scanning confocal microscope (Leica, Wetzlar, Germany).

### Statistical Analysis

Data were expressed as mean ± standard error (SEM) and analyzed with GraphPad Prism 6.0 statistical software (GraphPad Software, Inc., La Jolla, CA, USA). The significance of the differences between the two groups of mice was determined by an unpaired *t*-test. The level of significance was set at *p* < 0.05.

## Results

### Spatial Memory Impairment

**Figure 1:**
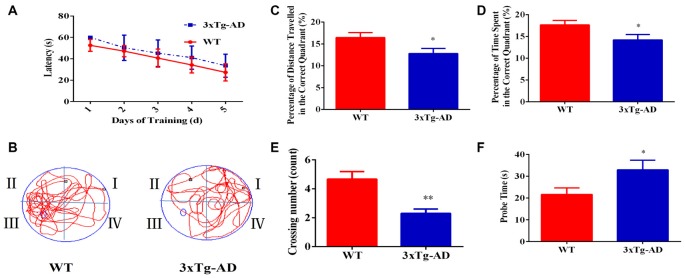
The spatial learning and memory ability of 3xTg-AD mice and wild-type (WT) mice. For the navigation test, the escape latency was measured to determine the spatial learning ability of 15-month-old male 3xTg-AD vs. age and sex-matched WT mice. **(A)** Escape latencies in the hidden-platform test. **(B)** Probe time showing representative swimming paths. **(C)** The percentage of distance traveled in the target quadrant. **(D)** The percentage of time spent in the target quadrant. **(E)** Numbers of crossings from the previous platform location; **(F)** probe time (s). *p < 0.05 and **p< 0.01 vs. the control mice (Mean ± standard error (SEM), *n* = 8–12 animals per group).

The Morris Water Maze test was used to investigate the spatial learning and memory abilities of 3xTg-AD vs. WT mice. 3xTg-AD mice showed a trend of mild learning impairment in the training session, although we did not observe a significant difference (Figure 1A). Seven days after the acquisition period, the probe trial was performed to evaluate the spatial memory of 3xTg-AD mice. Compared with the WT mice, the percentage of time spent and the percentage of distance traveled in the correct quadrant were significantly lower in 3xTg-AD mice (Figures 1B–D). Additionally, trends of increased probe time and decreased platform crossings were observed for 3xTg-AD relative to WT mice (Figures 1E,F). Learning and memory abilities were thus significantly impaired in the mouse model of AD.

### Increased Accumulation of Intracellular Aβ_1–42_

We used Aβ_1–42_, which induces pathological cleavage, phosphorylation and aggregation of full-length tau in cell systems (Hu et al., 2014), as a tissue biomarker of AD pathogenesis. Positive immunofluorescence for intracellular Aβ_1–42_ was significantly increased in entorhinal cortex, hippocampal CA1, CA3 and dental gyrus (DG) of 3xTg-AD mice compared to WT mice (Supplementary Figure S1).

### DNA Oxidative Damage

Immunofluorescent staining revealed that 8-OHdG immunoreactivity was markedly greater in entorhinal cortex, hippocampal CA1, CA3 and DG of 3xTg-AD mice compared to WT mice (Supplementary Figure S2). These data suggested that relative to WT animals, 3xTg-AD mice had more DNA oxidative damage in the brain.

### Differentially Expressed Hippocampal Mitochondrial and Nuclear Proteins

**Figure 2:**
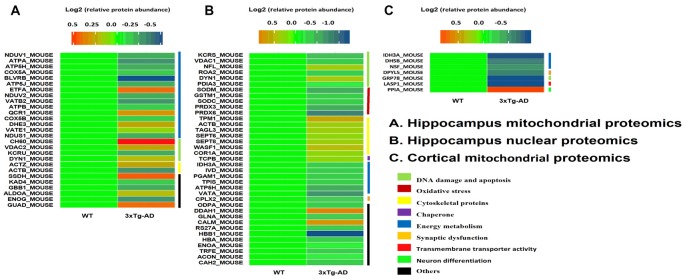
Hippocampal mitochondrial and nuclear protein spots differentially expressed between 3xT-AD mice and WT mice and identified by two-dimensional fluorescence difference gel electrophoresis (2D-DIGE)/MALDI-TOF-MS/MS. **(A,B)** Twenty-seven mitochondrial proteins and 37 nuclear proteins were differentially expressed between 3xTg-AD and WT mice. **(C)** Seven cortical mitochondrial proteins were differentially expressed in 3xTg-AD Mice vs. WT Mice (*n* = 6 per group). Proteins included those involved in energy metabolism, synaptic function, DNA oxidative damage and apoptosis, oxidative stress and cytoskeletal integrity.

We used 2D-DIGE and MALDI-TOF-MS/MS to analyze proteins in mitochondria and nuclear fractions prepared from the hippocampus of 3xTg-AD and WT mice. Separate runs were carried out for the hippocampal region and the entorhinal cortex. Representative 2D-DIGE gel images of hippocampal mitochondrial and nuclear proteins are shown in Supplementary Figures S3A–D, S4A–D, S5A–D. Protein spots with at least 1.1-fold and a *p*-value < 0.05 were considered differentially expressed and selected for MS/MS identification (Supplementary Figures S3E, S4E, S5E). Based on data obtained from the SwissProt database, differentially expressed proteins in hippocampal tissues are shown in Figure 2. Protein identification relied on at least two different peptide sequences and multiple peptide hits corresponding to every MS/MS event.

### Differentially Expressed Hippocampal Mitochondrial Proteins 3xTg-AD Mice vs. WT Mice

Twenty-seven mitochondrial protein spots were differentially expressed between 3xTg-AD and WT mice (Figure 2A). Among these proteins, 11 protein spots were increased and 16 decreased in 3xTg AD vs. WT mice.

There was substantially reduced relative abundance in 3xTg-AD mitochondria of biliverdin-IX beta reductase (BLVRB), a protein required for heme oxidation that releases free iron and is associated with the α-synuclein expression that accumulates in Parkinson disease (Scherzer et al., 2008) and other neurodegenerative disorders, including AD (Moussaud et al., 2014). Markedly higher protein abundance in hippocampal mitochondrial fractions of 3xTgAD vs. WT mice was seen for: (a) heat shock protein 60 (HS60), a molecular chaperone that maintains mitochondrial oxidative phosphorylation and tricarboxylic acid cycle (TCA) enzyme functionality against amyloid stress (Veereshwarayya et al., 2006; Mangione et al., 2016) but also mediates translocation of APP to mitochondria in 3xTg-AD cells and human AD tissue (Walls et al., 2012); (b) guanine deaminase (GUAD), which may play a role in microtubule assembly and axon restructuring (Yamatani et al., 2010); (c) electron transfer flavoprotein alpha subunit (EFTA), which participates in catalyzing the initial step of mitochondrial fatty acid beta-oxidation (GeneCards, LifeMap Sciences); and (d) succinate-semialdehyde dehydrogenase (SSDH), a key enzyme in the γ-aminobutyric acid (GABA) shunt, an alternative energy production pathway activated during cellular stress from reduced blood perfusion and resulting hypoxia in AD, when the TCA cycle is compromised (Salminen et al., 2016).

**Figure 3:**
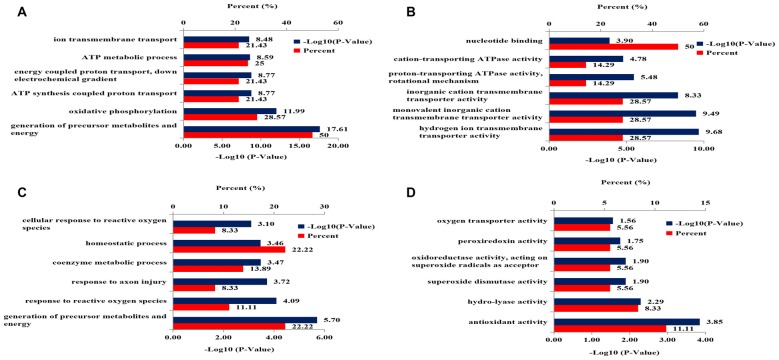
DAVID Gene Ontology enrichment analysis for the dysregulated mitochondrial proteins and nucleoproteins in hippocampus of 3xTg-AD mice. **(A)** Enrichment analysis for mitochondrial proteomics by biological processes. **(B)** Enrichment analysis for mitochondrial proteomics by molecular function. **(C)** Enrichment analysis for nuclear proteomics by biological process. **(D)** Enrichment analysis for nuclear proteomics by molecular function.

Gene ontology analysis was performed to categorize the differentially expressed mitochondrial proteins in the hippocampus of 3xTg-AD mice by biological processes and molecular functions. Biological processes revealed strong enrichment of generation of precursor metabolites and energy process, oxidative phosphorylation process, ATP synthesis-coupled proton transport process, energy-coupled proton transport process, ATP metabolic process, and ion transmembrane transport process (Figure 3A). Molecular function annotation revealed strong enrichment of: hydrogen ion transmembrane transporter activity, monovalent inorganic cation transmembrane transporter activity, inorganic cation transmembrane transporter activity, proton-transporting ATPase activity, cation-transporting ATPase activity and nucleotide binding (Figure 3B).

### Differentially Expressed Cortical Mitochondrial Proteins in 3xTg-AD Mice vs. WT Mice

Seven mitochondrial protein spots were differentially expressed in entorhinal cortex between 3xTg-AD and WT mice (Figure 2C). One protein spot was increased and six were decreased in 3xTg-AD vs. WT mice.

Protein phosphatase 1 catalytic subunit alpha (PP1A), a hippocampal neuronal protein implicated in neurite growth and the suppression of learning and memory and a potential mediator of cognitive decline during ageing (Genoux et al., 2002; Buchser et al., 2010), showed increased relative protein abundance. Reduced relative abundance was found for: (a) LASP1 (Lim and SH3 protein 1), an actin-binding neuronal protein concentrated at post-synapses and dendritic spines in the hippocampus (Phillips et al., 2004), downregulated by the NMDA receptor antagonist MK801 and, in single-nucleotide polymorphic form, implicated in susceptibility to schizophrenia (Joo et al., 2013); (b) isocitrate dehydrogenase 3 (IDH3A), a TCA cycle enzyme responsible for oxidative decarboxylation of isocitrate to 2-oxoglutarate; (c) glucose-regulated protein 78 (GRP78), a major ER chaperone and central regulator for ER stress and unfolded protein response with anti-apoptotic properties (Wang M. et al., 2010); (d) succinate dehydrogenase iron-sulfur subunit (DHSB), a membrane-bound FAD-containing TCA-cycle enzyme responsible for fumarate-succinate interconversion in aerobic cell growth (UniProt); (e) dihydropyrimidinase-related protein 5 (DPYL5), which may function in neuronal differentiation and/or axon growth (PhosphoSitePlus, Cell Signalling Technology); and (f) NSF attachment protein alpha (NSF), a member of the SNAP family that plays a critical role in the docking and fusion of vesicles to target membranes as part of the NSF-SNAP-SNARE complex (Nishizaki, 2016) and that is enriched in a mouse motor-neuron cell-like model of ALS (Kim et al., 2017).

### Differentially Expressed Hippocampal Nuclear Proteins in 3xTg-AD Mice vs. WT Mice

Thirty-seven nuclear protein spots were differentially expressed between 3xTg-AD and WT mice (Figure 2B). Among these proteins, 12 protein spots were increased and 25 decreased in 3xTg-AD relative to WT mice.

Prominent relative increased protein abundance was seen for: (a) calmodulin (CALM), a major neuronal calcium signaling protein that is deranged in AD (Popugaeva et al., 2017) and has a very high affinity for neurotoxic Aβ peptides resulting in reduced pathologic Aβ fibrillation (Corbacho et al., 2017); (b) dimethylargininase-1 (DDAH1), overexpression of which promotes asymmetric dimethylarginine degradation and significantly attenuates oxidative stress and Aβ secretion in human SH-SY5Y cells overexpressing (as in 3xTg-AD mice) the Swedish mutant form of human Aβ precursor protein (Luo et al., 2015). The greatest reduction in 3xTg-AD nuclei relative protein abundance was that of hemoglobin subunit 1 beta (HBB1), which participates in iron and oxygen binding and in oxygen transport; to a lesser degree, in the peroxiredoxins PRDX6 and PRDX3, key players in cellular redox function and protection against oxidative injury. Modestly reduced relative abundance was found for: (a) mitochondrial enzyme superoxide dismutase (SOD) 2 (Fe, Mn), which destroys toxic superoxide anion radicals, participates in oxygen binding and is linked to neurodegenerative disease (Flynn and Melov, 2013); (b) ATPase H^+^ transporting v1 subunit A (VATA), a molecular partner of the ER transmembrane Wolfram syndrome 1 (WFS1) protein, which regulates its expression and stability and thereby participates in the pathogenesis of the neurodegeneration and diabetes mellitus seen in Wolfram autosomal recessive disease (Gharanei et al., 2013); and (c) ATP synthase subunit d (ATP5H), which has been associated with Aβ toxicity (Mukherjee et al., 2017).

Gene ontology analysis was performed to functionally categorize the differentially expressed nucleoproteins in hippocampus of 3xTg-AD mice by biological processes and molecular functions. The results for biological processes revealed strong enrichment for: generation of precursor metabolites and energy process, response to reactive oxygen species (ROS), response to axon injury process, coenzyme metabolic process, homeostatic process and cellular response to ROS (Figure 3C). Molecular function annotation revealed strong enrichment of antioxidant activity, hydro-lyase activity, SOD activity, oxidoreductase activity, peroxiredoxin activity and oxygen transporter activity (Figure 3D).

### STRING Analysis for the Differential Mitochondrial and Nuclear Proteins

**Figure 4:**
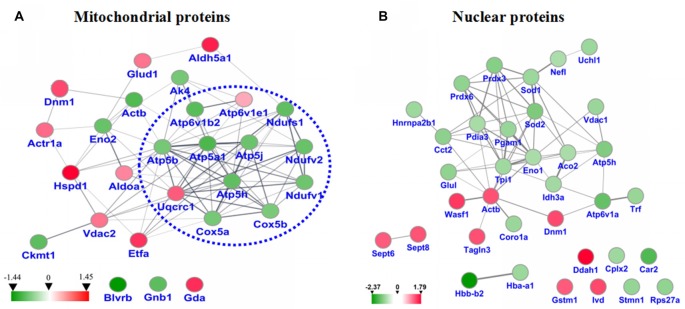
**(A)** Protein-protein interaction network among the dysregulated proteins of hippocampal mitochondria. **(B)** Protein-protein interaction network among the dysregulated proteins of hippocampal nuclei. Balls represent proteins, and lines represent interactions between proteins, some of which are increased (red arrows) and others decreased (green arrows) in 3xTg-AD vs. WT mouse hippocampus. Thick, thin and dashed lines indicate high-, medium- and low-confidence interactions. Interactions between hippocampal proteins showing increased and reduced abundance in 3xTg-AD vs. WT mice were identified by DIGE-based proteomics and analyzed with the STRING database.

**Figure 5:**
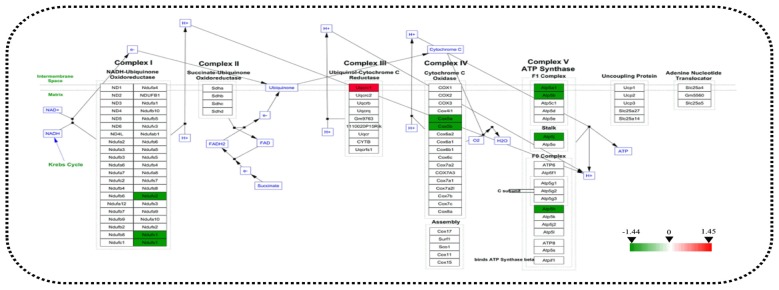
Analysis of altered proteins targeting the ATP-generation pathway. Dysregulated mitochondrial proteins associated with the electron transport chain (ETC). All identified hippocampal protein changes in 3xTg-AD vs. WT mice were mapped to their related WikiPathway based on the published database. Boxes are labeled with the protein name.

STRING analysis was performed to reveal the protein-protein interaction networks among the differential proteins in 3xTg-AD mice. Interactions between proteins related to energy metabolism were evident, such as NADH-ubiquinone oxidoreductase 75 kDa subunit (NDUS1), NADH dehydrogenase [ubiquinone] flavoprotein 1 (NDUV1), ATP synthase subunit alpha (ATPA), ATP5H, cytochrome c oxidase subunit 5A (COX5A), ATP synthase-coupling factor 6 (ATP5J), NADH dehydrogenase [ubiquinone] flavoprotein 2 (NDUV2), ATP synthase subunit beta (ATPB) and cytochrome b-c1 complex subunit 1 (QCR1; Figure 4A). Interactions among dynamin-1 (DYN1), actin, cytoplasmic 1 (ACTB), and alpha-centractin (ACTZ) were also found (Figures 4A,B). In addition, WikiPathway analysis revealed that NDUV1, NDUV2, NDUS1, QCR1, COX5A, COX5B, ATPA, ATPB, ATP5H, ATP5J are located in the ETC Complexes I and III-V (Figure 5).

### Two Proteins Abnormally Expressed Both in Hippocampal Mitochondria and Nuclei

**Figure 6:**
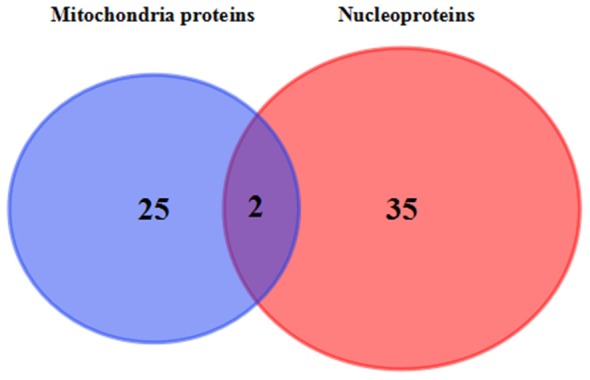
Commonly differentially expressed proteins in hippocampal mitochondria and nuclei. Venn diagram showing the identity of hippocampal tissue proteins found in both mitochondrial and nuclear fractions of 3xTg-AD vs. WT mice. The two commonly differentially expressed proteins are involved in the ETC (QDCX2) and synaptic function and apoptosis (P39052).

Venn diagram showed that two identical proteins were abnormally expressed both in mitochondrial and nuclei fractions of 3xTg-AD mouse hippocampus, namely: mitochondrial division-regulated protein DYN1 and ETC-related ATP5H (Figure 6). DYN1 was significantly increased in the hippocampal mitochondrial and nuclear pellet (Table 1).

**Table 1:** The detailed list of commonly differentially expressed proteins in hippocampal mitochondria and nuclei.

Uniprot no.	Protein name	Ratio	
		Mitochondria	Nuclear
P39053	Dynamin-1	1.23	1.33
Q9DCX2	ATP synthase subunit d, mitochondrial	−1.18	−1.43

### Representative Protein Spots Differentially Expressed in 3xTg-AD vs. WT Mice

**Figure 7:**
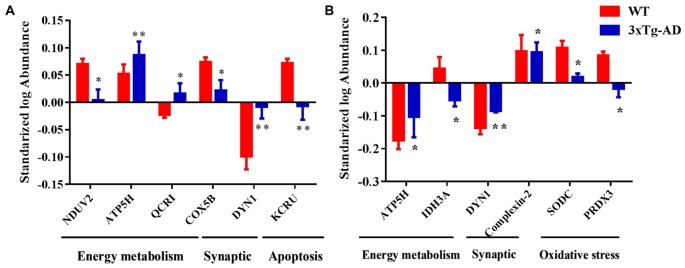
Representative protein spots differentially expressed in 3xTg-AD mice. Protein spots abnormally expressed in **(A)** hippocampal mitochondria and **(B)** hippocampal nuclei. *p < 0.05 and **p < 0.01 vs. the control mice (Mean ± SEM, *n* = 3–6 for each group).

Based on the results of functional analysis, we selected seven representative protein spots in hippocampal mitochondria and six representative protein spots in hippocampal nuclei for fluorescence intensity analysis (Figures 7A,B). The representative mitochondrial proteins are: ETC-related proteins NDUV2, ATP5H, COX5B, UCRI; apoptosis-related proteins DYN1, creatine kinase U-type (KCRU), respectively (Figure 7A). The representative nuclear proteins are: energy metabolism-related proteins ATP5H, IDH3A; apoptosis-related protein DYN1; synaptic-related protein complexin-2; oxidative stress-related proteins SODC, PRDX3, respectively (Figure 7B).

## Discussion

Mounting evidence suggests that mitochondrial dysfunction is closely related to the pathogenesis of several neurodegenerative disorders, including AD (Müller et al., 2010; Calkins et al., 2011; Selim and Hassaan, 2017; Weng et al., 2017). Mitochondrial dysfunction is mainly manifested as an energy metabolism disorder, ETC damage, mitochondrial dynamics disorder and related descriptors (Minoshima et al., 1997; Etminan et al., 2005; Chan, 2006b; Aung et al., 2017). We sought to illuminate specific molecular mechanisms underlying AD-related mitochondrial dysfunction with the aid of a well-accepted 3xTg-AD mouse model of the human disease at a life stage (15 months old) when brain neuronal degeneration is well underway. Consequently, the learning and memory ability of animals was significantly impaired; hippocampal Aβ_1–42_ immunofluorescence was abnormally prominent, and DNA oxidative damage was increased. Actually, 3xTg-AD mice showed a trend of mild learning impairment in the training session, although we did not observe significant differences.

Proteomics analysis of hippocampal subcellular fractions revealed that the murine AD-related behavioral and pathological changes were coincident with significant differential expression of 27 mitochondrial proteins and 37 nuclear proteins. These proteins were involved in energy metabolism, synaptic dysfunction, DNA damage and apoptosis, oxidative stress, and cytoskeletal integrity. Among them, mitochondrial division-regulated protein DYN1 and ETC-related protein ATP5H were significantly increased and decreased, respectively, suggesting that hippocampal neurons in AD mice suffer dysfunction of energy metabolism and mitochondrial dynamics.

### Mitochondrial Respiration and Metabolism

Studies have shown that AD pathogenesis is accompanied by abnormal energy metabolism (Hartl et al., 2012; Dixit et al., 2017). Indeed, among the differential mitochondrial proteins, we found that more than half (55.6%) was involved in energy metabolism. In particular, 10 abnormally expressed proteins were involved in the mitochondrial respiratory chain, including: NDUV1, NDUV2 and NDUS1 in Complex I; UCRI in Complex III; COX5A, COX5B in Complex IV, and ATP5A, ATP5B, ATP5H and ATP5J in Complex V (Mitchell, 1961; Koopman et al., 2013). Interestingly, 10 of the ETC-related proteins are encoded by nuclear genes, not mitochondrial genes (Mastroeni et al., 2017). A previous study of whole-brain mitochondrial fractions of 12-month-old female 3xTg-AD mice found abnormal State 3 and 4 oxygen consumption, lower Complex IV activity and elevated oxidative stress accompanied by increased Aβ and γ-secretase components (Walls et al., 2012). The activity of mitochondrial Complexes I-IV is decreased in AD brain, which results in an increase of ROS (Atamna and Frey, 2007). Collectively, it is evident that perturbations of mitochondrial energy metabolism-related proteins responsible for ATP generation via oxidation phosphorylation (OXPHOS; Saraste, 1999; Newmeyer and Ferguson-Miller, 2003), especially nuclear-encoded OXPHOS proteins, play an important, perhaps central, role in the development of AD.

### DNA Damage and Apoptosis-Related Proteins

DYN1 plays an important role in mitochondrial fission and is essential for the distribution of mitochondria in axons, dendrites and synapses (Wang et al., 2008; Chen and Chan, 2009; Wang X. et al., 2010; Reddy et al., 2011; Roy et al., 2015). The protein is concentrated in presynaptic terminals (Gray et al., 2003) where it participates in synaptic vesicle recycling, and its reduced expression has been associated with impaired spatial memory (Magarinos et al., 1997). Studies have shown that DYN1 interacts with Aβ and phosphorylated tau, which may lead to excessive mitochondrial fragmentation, mitochondrial and synaptic defects that may eventually cause neuronal damage and cognitive decline (Manczak et al., 2011; Manczak and Reddy, 2012; Shirendeb et al., 2012). Furthermore, reduced DYN1 can prevent Aβ and phosphorylation of tau-induced mitochondrial dysfunction and synaptic damage in AD (Kandimalla et al., 2016; Manczak et al., 2016). In addition, DYN1 and mitofusion 2 protein (Mfn2) play an important role in regulating mitochondrial morphology and neuronal viability compared with non-neuronal cells (Uo et al., 2009). In sum, DYN1 has an important role in AD and other neurodegenerative diseases. The significant increase of DYN1 in 3xTg-AD vs. WT mice suggests a role in AD linked to the function and distribution of mitochondria.

### Oxidative Stress

Oxidative stress is also considered to be an important factor in AD (Mamelak, 2007). SOD functions to reduce oxidative stress by converting the superoxide byproducts of oxidative phosphorylation to hydrogen peroxide and diatomic oxygen (Zhao et al., 2005). Additionally, the H_2_O_2_-scavenging peroxiredoxins (Prxs) have an important role in the regulation of ROS (Kim et al., 2016). Previous studies demonstrate that Prx1 and Prx6 overexpression reduce AD pathology; Prx2 protects hippocampus cognitive function from age-related oxidative stress, and Prx5 has a protective effect on neurons (Krapfenbauer et al., 2003; Chua et al., 2010; Kim et al., 2011, 2013; Lee et al., 2011; Zhao and Wang, 2012; De Simoni et al., 2013). We found that protein levels of SOD [Mn] (SODM), SOD [Cu-Zn] (SODC), peroxiredoxin-3 and peroxiredoxin-6 were significantly decreased in nuclei of 3xTg-AD vs. WT mice, results that suggest these four proteins are involved in an oxidative stress-mediated process in the hippocampus of AD mice. Additionally, 5 oxidative stress-related proteins and one synaptic dysfunction-related protein (complexin-2) were significantly decreased in the hippocampal nuclei of 3xTg-AD relative to WT mice.

### Synaptic Dysfunction

Complexin-2 is an important synaptic plasticity and neurotransmitter-release regulator (Reim et al., 2001; Hill et al., 2006). The protein is significantly decreased in the hippocampus of AD patients (Tannenberg et al., 2006), and we reported previously that the expression of complexin-2 is decreased in the brains of AD mice (Yu et al., 2015; Ying et al., 2017). Furthermore, complexin-2 knockout mice show prominent cognitive impairment (Glynn et al., 2003). In the present study, we found complexin-2 to be significantly decreased in hippocampal nuclei of 3xTg-AD vs. WT mice, indicating that changes in complexin-2 (as well as DYN1) expression contribute to the synaptic dysfunction that probably underlies the spatial memory impairment of AD mice.

## Conclusion

**Figure 8:**
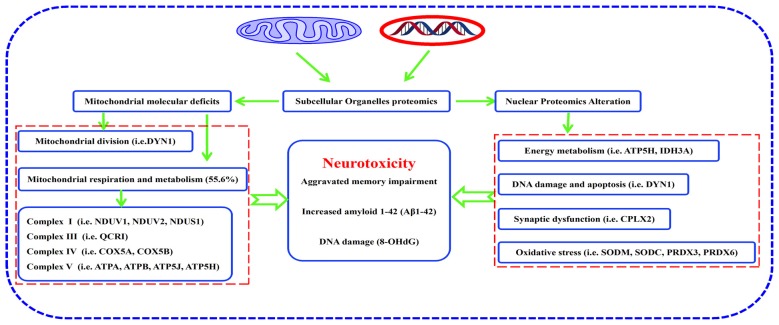
Pathway from hippocampal molecular defects to AD neurotoxicity. Proteomic analysis of subcellular organelles showed that many proteins were differentially expressed in mitochondria and nuclei, including ETC-related proteins, mitochondrial division-regulated proteins, oxidative stress-related proteins, and synaptic-related proteins, which in concert, correlate with neurodegeneration in the 3xTg-AD mouse model of Alzheimer disease. Protein dynamin-1 (DYN1) is a candidate for an early biomarker of AD.

In summary, many proteins were differentially expressed in hippocampal mitochondria and nuclei of mature, triple AD-transgenic mice displaying spatial learning and memory impairment underpinned by human AD-like neurodegeneration. These proteins included: ETC-related proteins, such as NDUV1, NDUV2, NDUS1, UCRI, COX5A, COX5B, ATP5A, ATP5B, ATP5H and ATP5J, mitochondrial division-regulated protein DYN1, oxidative stress-related proteins SODM, SODC, peroxiredoxin-3, and peroxiredoxin-6, synaptic-related protein complexin-2. Reduced expression of DYN1 implicates mitochondrial distribution as well as presynapse integrity in synaptic dysfunction. Taken together, the molecular changes and associated biological/pathogenic processes, particularly involving respiratory chain-related proteins and mitochondrial division-regulated protein DYN1, may represent novel biomarkers of AD that contribute to neurodegeneration in this disease (Figure 8). Study of the temporal evolution of AD-related changes in the brains of 3xTg-AD mice of different ages are now needed to determine whether these proteins represent early markers of the progressive neurodegenerative process.

## Author Contributions

HY, XL and DW performed the experiments, analyzed the data and drafted the manuscript. ZZ, YG, XR, BX, JY, JL and XY designed the study and analyzed the data. PSS, J-ZW and XY wrote and revised the manuscript.

## Conflict of Interest Statement

The authors declare that the research was conducted in the absence of any commercial or financial relationships that could be construed as a potential conflict of interest.

## Footnotes

^1^https://david-d.ncifcrf.gov/

^2^https://string-db.org/

^3^http://www.pangloss.com/seidel/Protocols/venn.cgi

## Supplementary Material

FIGURE S1 Increased intracellular Aβ_1–42_ in 3xTg-AD mice. **(A)** Brain sections containing cortex, hippocampal CA1, CA3 and DG regions were stained with anti-Aβ_1–42_ antibody to detect the level of intracellular Aβ_1–42_. Representative images were selected from 3xTg-AD mice and WT mice. **(B)** Quantitative analysis of Aβ_1–42_-positive neurons in cortex. **(C)** Quantitative analysis of Aβ_1–42_-positive neurons in CA1. **(D)** Quantitative analysis of Aβ_1–42_-positive neurons in CA3. **(E)** Quantitative analysis of Aβ_1–42_-positive neurons in DG. ***p* < 0.01 and ****p* < 0.001 vs. the control mice (Scale bar = 100 μm, *n* = 4 for each group).

FIGURE S2 Increased 8-OHdG in hippocampus of the 3xTg-AD mice. **(A)** Brain sections containing cortex, hippocampal CA1, CA3 and DG regions were stained with anti-8-OHdG antibody to detect the level of oxidative DNA damage. Representative images were selected from 3xTg-AD mice and WT mice. **(B)** Quantitative analysis of 8-OHdG-positive neurons in cortex. **(C)** Quantitative analysis of 8-OHdG-positive neurons in CA1. **(D)** Quantitative analysis of 8-OHdG-positive neurons in CA3. **(E)** Quantitative analysis of 8-OHdG-positive neurons in DG. **p* < 0.05 vs. the control mice (Scale bar = 100 μm, *n* = 4 for each group).

FIGURE S3 A representative 2D-DIGE gel image of hippocampal mitochondrial proteins from 3xTg-AD mice and WT mice. Hippocampal mitochondrial proteins from 3xTg-AD mice and WT mice were labeled with Cy3 or Cy5 dyes, respectively (*n* = 6 for each group). An internal standard protein sample (a mixture of all mitochondria samples) was labeled with the Cy2 dye. The CyDye-labeled samples were combined, and the proteins were co-separated in the first dimension via IEF in 24 cm pH 3–11 nonlinear IPG strips, followed by separation in the second dimension via SDS-PAGE. Spots of interest were manually excised, digested and subjected to identification by MALDI-TOF-MS/MS. **(A)** Cy2-labeled proteins as internal standards. **(B)** Cy3-labled hippocampal mitochondrial proteins of 3xTg-AD mice. **(C)** Cy5-labeled hippocampal mitochondrial proteins of WT mice. **(D)** A merged image showing Cy2-, Cy3- and Cy5-labeled proteins. **(E)** Grayscale 2D-DIGE gel image showing 27 differentially expressed protein spots identified by MALDI-TOF-MS/MS (black numbers in white squares) in hippocampal mitochondria of 3xTg-AD mice compared with WT mice.

FIGURE S4 A representative 2D-DIGE gel image of hippocampal nuclear proteins from 3xTg-AD mice and WT mice. Hippocampal nuclear proteins from 3xTg-AD mice and WT mice were labeled with Cy3 or Cy5 dyes, respectively (*n* = 6 for each group). An internal standard protein sample (a mixture of all nucleus samples) was labeled with the Cy2 dye. The CyDye-labeled samples were combined, and the proteins were co-separated in the first dimension via IEF in 24 cm pH 3–11 nonlinear IPG strips, followed by separation in the second dimension via SDS-PAGE. Spots of interest were manually excised, digested and subjected to identification by MALDI-TOF-MS/MS. **(A)** Cy2-labeled proteins as internal standards. **(B)** Cy3-labled hippocampal nuclear proteins of 3xTg-AD mice. **(C)** Cy5-labeled hippocampal nuclear proteins of WT mice. **(D)** Merged image showing Cy2-, Cy3- and Cy5-labeled proteins. **(E)** Grayscale 2D-DIGE gel image showing 37 differentially expressed protein spots identified by MALDI-TOF-MS/MS (black numbers in white squares) in the hippocampal nucleus of 3xTg-AD mice compared with WT mice.

FIGURE S5 A representative 2D-DIGE gel image of cortical mitochondrial proteins from 3xTg-AD mice and WT mice. Cortical mitochondrial proteins from 3xTg-AD mice and WT mice were labeled with Cy3 or Cy5 dyes, respectively (*n* = 6 for each group). An internal standard protein sample (a mixture of all mitochondria samples) was labeled with the Cy2 dye. The CyDye-labeled samples were combined, and the proteins were co-separated in the first dimension via IEF in 24 cm pH 3–11 nonlinear IPG strips, followed by separation in the second dimension via SDS-PAGE. Spots of interest were manually excised, digested and subjected to identification by MALDI-TOF-MS/MS. **(A)** Cy2-labeled proteins as internal standards. **(B)** Cy3-labled cortical mitochondrial proteins of 3xTg-AD mice. **(C)** Cy5-labeled cortical mitochondrial proteins of WT mice. **(D)** A merged image showing Cy2-, Cy3- and Cy5-labeled proteins. **(E)** Grayscale 2D-DIGE gel image showing 7 differentially expressed protein spots identified by MALDI-TOF-MS/MS (black numbers in white squares) in cortical mitochondria of 3xTg-AD mice compared with WT mice.
