# Supplementary figures and images for: Mitochondrial Molecular Abnormalities Revealed by Proteomic Analysis of Hippocampal Organelles of Mice Triple Transgenic for Alzheimer Disease
Source: Front Mol Neurosci. 2018 Mar 9;11:74. doi: 10.3389/fnmol.2018.00074 (PMC5854685; doi:10.3389/fnmol.2018.00074)

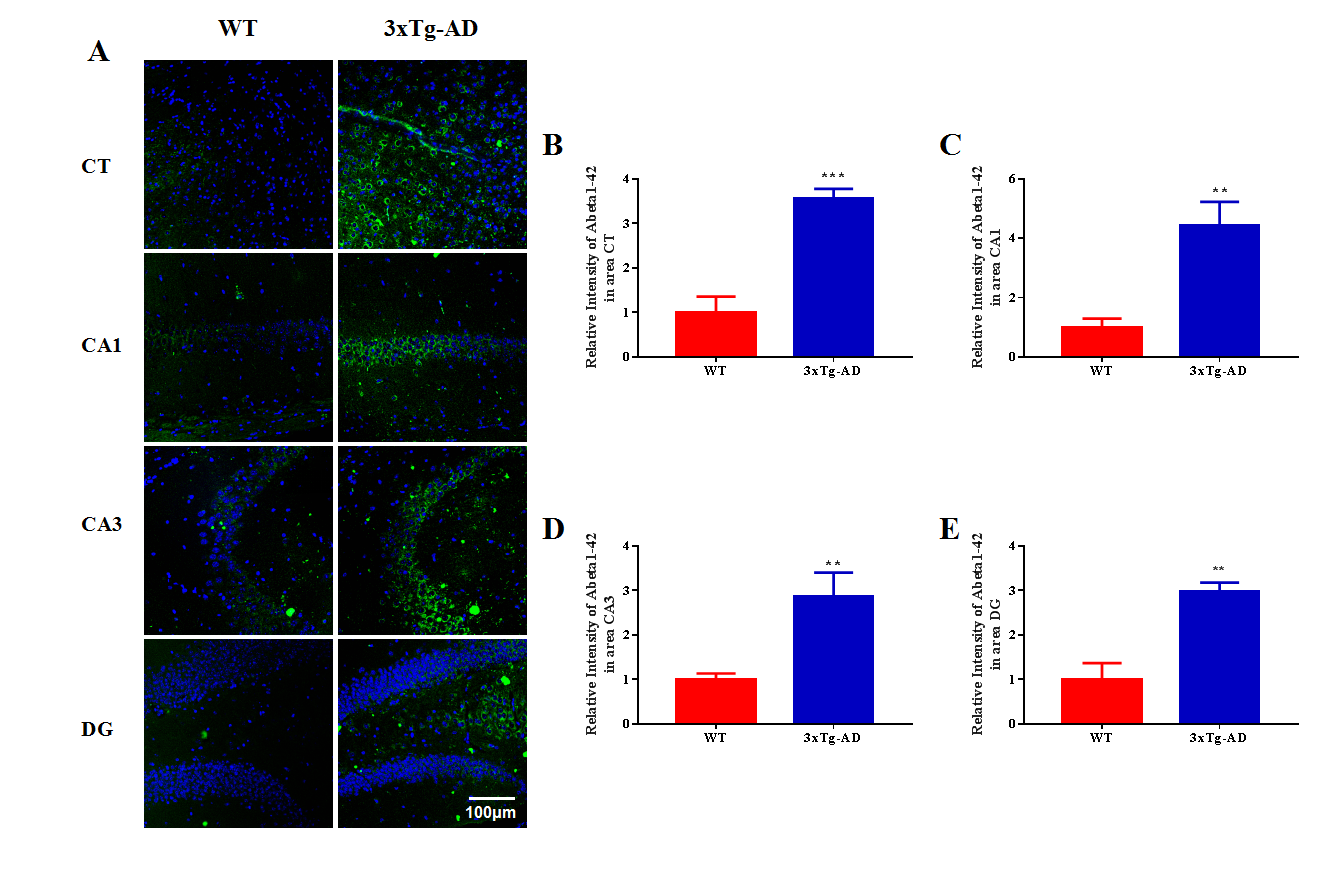

Supplement: FIGURE S1 — Increased intracellular Aβ1–42 in 3xTg-AD mice. (A) Brain sections containing cortex, hippocampal CA1, CA3 and DG regions were stained with anti-Aβ1–42 antibody to detect the level of intracellular Aβ1–42. Representative images were selected from 3xTg-AD mice and WT mice. (B) Quantitative analysis of Aβ1–42-positive neurons in cortex. (C) Quantitative analysis of Aβ1–42-positive neurons in CA1. (D) Quantitative analysis of Aβ1–42-positive neurons in CA3. (E) Quantitative analysis of Aβ1–42-positive neurons in DG. **p < 0.01 and ***p < 0.001 vs. the control mice (Scale bar = 100 μm, n = 4 for each group). [file Image_1.TIF]

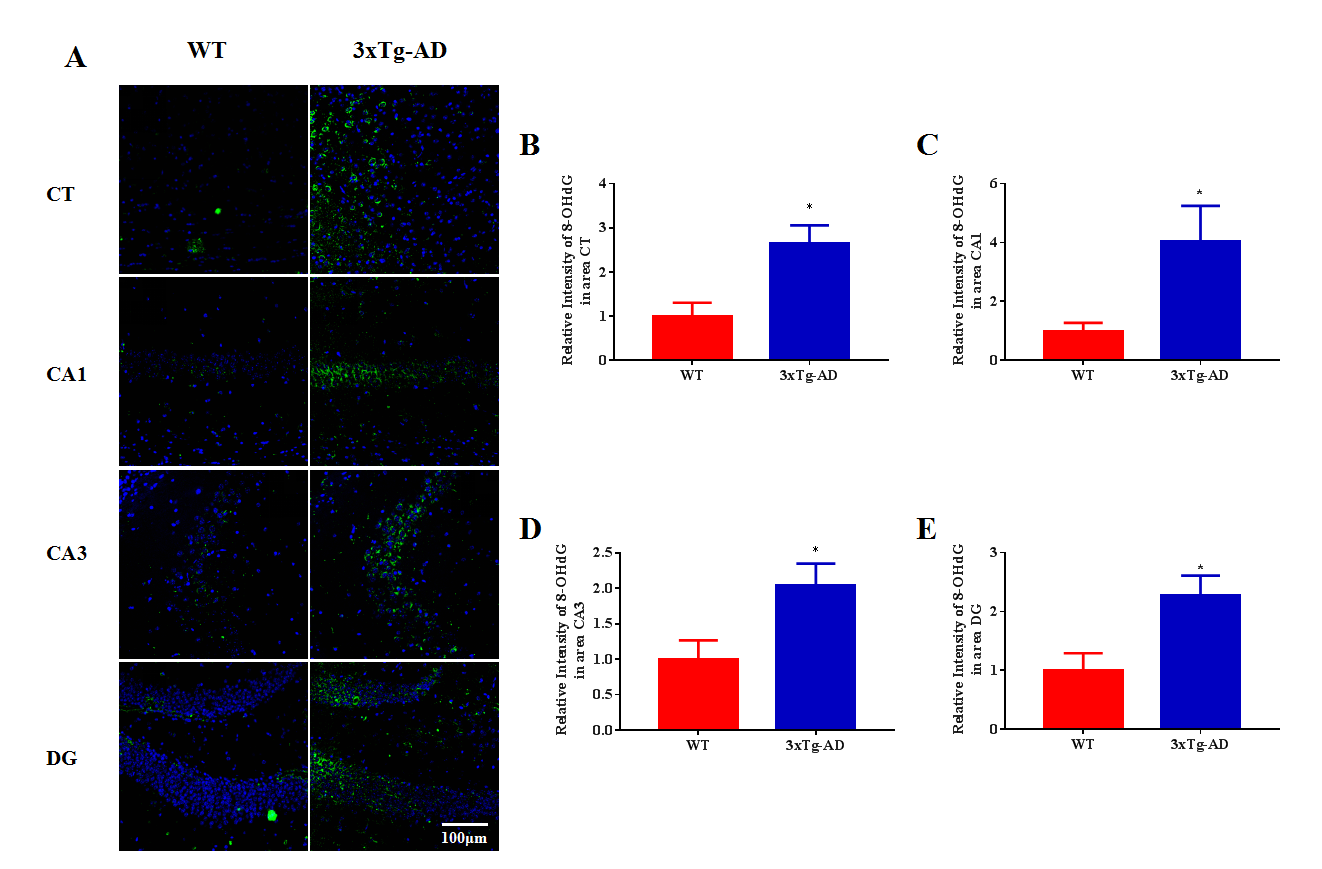

Supplement: FIGURE S2 — Increased 8-OHdG in hippocampus of the 3xTg-AD mice. (A) Brain sections containing cortex, hippocampal CA1, CA3 and DG regions were stained with anti-8-OHdG antibody to detect the level of oxidative DNA damage. Representative images were selected from 3xTg-AD mice and WT mice. (B) Quantitative analysis of 8-OHdG-positive neurons in cortex. (C) Quantitative analysis of 8-OHdG-positive neurons in CA1. (D) Quantitative analysis of 8-OHdG-positive neurons in CA3. (E) Quantitative analysis of 8-OHdG-positive neurons in DG. *p < 0.05 vs. the control mice (Scale bar = 100 μm, n = 4 for each group). [file Image_2.TIF]
